# Impact of language concordant care on comprehension and adherence to infection prevention protocols among hospitalized patients: A multicentre survey

**DOI:** 10.14745/ccdr.v52i04a05

**Published:** 2026-04-30

**Authors:** Paige Reason, Amna Rizvi, Radhika Chawla, Sheila Le-Abuyen, Vishnuka Arulsundaram, Hassen Nandwani, Alon Vaisman, Jerome A Leis

**Affiliations:** 1 Sunnybrook Health Sciences Centre, Toronto, ON; 2 University Health Network, Toronto, ON; 3 Temerty Faculty of Medicine, University of Toronto, Toronto, ON; 4 Centre for Quality Improvement and Patient Safety, University of Toronto, Toronto, ON

**Keywords:** language, infection, equity, prevention, healthcare

## Abstract

**Background:**

Language concordant care can influence patient outcomes. The level of language concordant care and its impact on adherence to infection prevention protocols has not been well described.

**Objective:**

To assess whether patients’ preferred language impacts their ability to follow infection prevention protocols.

**Methods:**

A multicentre survey was performed to assess understanding and self-reported adherence to two different sets of infection prevention instructions: preoperative chlorhexidine bathing before cardiac surgery and central line care during chemotherapy for hematologic malignancy. Standardized interviews were conducted following cardiac surgery or central vascular catheter insertion. Preferred language was defined by patient’s stated preference, with professional hospital-based interpretation used for patients with non-English language preference.

**Results:**

Among 238 eligible patients, 222 (93%) completed the interview across both sites, including 179 (81%) with English preference and 43 (19%) with non-English preference. Comprehension of infection prevention instructions among English-preferred and non-English-preferred groups was no different (97.2% vs 93.0%; *p*=0.19), and similar for reported adherence (97.8% vs 97.7%; *p*=0.97). Among 26 (65%) non-English-preferred patients, instructions were reported to be interpreted by family rather than by hospital-based interpretation services. Qualitative themes included reliance on family for interpretation or other informal solutions, patient frustration with language barrier and expressed desire for improved language services.

**Conclusion:**

No significant difference was identified in comprehension or adherence to infection prevention protocols based on language preference; however, closing this gap relied on family members or other informal solutions. Given the risks and inequity of such workarounds, improved use of hospital-based interpretation services is needed.

## Introduction

Roughly one in five Canadians report that their first language is a language other than French or English ((1)). Language barriers during a hospital stay are associated with reduced satisfaction of patients and providers as well as inferior patient outcomes ((2–4)).

Language discordant care may also be a risk factor for development of some healthcare-associated infections. In a recent large retrospective cohort study in the United States, speaking a language other than English was associated with a 60% higher risk of central-line associated bloodstream infection ((5)).

In Canada, there is a paucity of data regarding whether patients receive language concordant care related to protocols intended to mitigate their risk of healthcare-associated infection ((6–8)). A multicentre survey was performed to assess whether there was a difference in comprehension and/or adherence to two infection prevention protocols based on a patient’s reported preferred language.

## Methods

This study was conducted at two large academic hospitals in Toronto among two distinct patient populations that receive standardized instructions to mitigate risk of infection. The first population included patients undergoing cardiac surgery and receiving instructions on preoperative bathing with chlorhexidine. The second population included patients with hematologic malignancies who receive instructions on how to reduce their risk of infection following outpatient central-line insertion.

Eligible participants from the cardiac surgery group were admitted patients who underwent elective cardiac surgery within the previous 14 days and had received preoperative instructions in the outpatient clinic. Eligible participants from the malignant hematology population included patients who had their first central-line inserted within the previous 60 days and had spent at least 24 hours at home since insertion. Those declining to be interviewed, or with cognitive impairment, or speaking an unavailable language for interpretation, or reporting never having received infection prevention instructions were excluded from both groups.

Between June 1, 2024 and October 31, 2024, eligible participants were approached by infection prevention and control professionals. Those consenting to participate underwent anonymous semi-structured interviews lasting 5–10 minutes. The interview first confirmed their preferred language and verified their understanding and reported adherence to specific infection prevention protocols (**Table 1**). If a patient identified a non-English language as their preferred language, the remainder of the interview was conducted using the hospital provided interpretation service using a speaker phone or in-person interpretation. An interview tool contained standardized prompts to assist patients with understanding the question as needed. A second interviewer was present for the first six non-English interviews (24 questions) to independently validate the quantitative responses across both patient populations (Kappa=1.0).

**Table 1:** Standardized interview questions to determine comprehension and reported adherence to infection prevention instructions

Cardiac surgery questions	Malignant hematology questions
**Quantitative questions**	
1. What is your preferred language? 2. Please describe the instructions you were given: What were you told about bathing/cleaning protocols when at home before your surgery? (can describe bathing with general details=1; otherwise=0)	1. What is your preferred language? 2. Please describe the instructions you were given: What were you told about how to care for your line while at home? (can describe at least two—e.g., keep dry, keep clean/covered, no pets, no underwater=1; fewer than two=0)
3. Prompts, only if scored zero: a. Were you told to use the yellow soap for five days prior to surgery? (yes with prompting=1; no with prompting=0)	3. Prompts, only if scored zero: a. Do you know if you are allowed to submerse your line in water? (neither PICC nor Hickman line can be fully submerged, yes=1; otherwise=0) b. Were you asked to keep your dressing dry? (yes with prompting=1; no with prompting=0)
4. (For patients with non-English preferred language) How were these instructions given to you? (no interpreter; interpretation by third party; interpretation by family/friend)	4. (For patients with non-English preferred language) How were these instructions given to you? (no interpreter; interpretation by third party; interpretation by family/friend)
5. Adherence to recommendations: Did you perform bathing before your surgery? (yes=1; no=0)	5. Adherence to recommendations: Did you keep your line clean, covered, and away from water? (yes=1; no=0)
**Qualitative questions**	
6. (For patients with non-English preferred language) During your interactions with the healthcare team, did you feel your ability to communicate in English influenced your ability to follow the recommendations?	6. (For patients with non-English preferred language) During your interactions with the healthcare team, did you feel your ability to communicate in English influenced your ability to follow the recommendations?
7. (For patients with non-English preferred language) Are there any other ways in which your ability to communicate in English affected your health in the hospital?	7. (For patients with non-English preferred language) Are there any other ways in which your ability to communicate in English affected your health in the hospital?

Abbreviation: PICC, peripherally inserted central catheter

The primary outcome of the study was the proportion of patients that could demonstrate understanding of infection prevention instructions. The secondary outcome was the proportion of patients that adhered to infection prevention instructions. Patients were categorized based on self-reported preferred language (English or non-English language). This categorization was used as English is the language used routinely in the two participating hospitals. French preference was analyzed with the non-English preference group as too few patients preferred French. If present, the type of method of interpretation used by non-English preferred patients was recorded. The differences in group proportions were compared using chi-square tests. Responses to open-ended interview questions (questions 6, 7) were summarized and reviewed for emerging qualitative themes using grounded theory methodology ((9)). Research ethics review was not required because the project met criteria for exemption based on institutional process for quality improvement.

## Results

Among 238 eligible patients across both sites, 222 (93.3%) consented to participate while 11 (4.6%) declined and five (2.2%) were excluded due to confusion (n=2), lack of available interpreter for language (n=1) and reporting not receiving infection prevention instructions (n=2). There were 179 (80.6%) participants with English language preference and 43 (19.4%) with non-English language preference.

The proportion of patients who confirmed comprehension and reported adherence to infection prevention protocols are summarized in **Table 2**. Overall, no significant difference was identified between English-preferred and non-English-preferred groups for either comprehension (97.2% vs 93.0%; *p*=0.19) or adherence (97.8% vs 97.7%; *p*=0.97). Among 26 (65%) non-English preferred patients, instructions were interpreted for them by a family member or other informal means, rather than by hospital-based interpretation service (**Table 3**). All three patients who did not comprehend the instructions had neither assistance from family nor informal means nor use of hospital-based interpretation.

**Table 2:** Proportion of patients confirming comprehension of and adherence to infection prevention instructions based on language preference

Outcome	Language preference	Hospital A	Hospital B	Combined	*p*-value
Comprehension	English	90 (98%)	84 (97%)	174 (97%)	0.19
	Non-English	20 (87%)	20 (100%)	40 (93%)	
Adherence	English	90 (98%)	85 (98%)	175 (98%)	0.97
	Non-English	23 (100%)	19 (95%)	42 (98%)	

**Table 3:** Comprehension of instructions by method of interpretation

Method of interpretation	Patient comprehended (n=40)	Patient did not comprehend (n=3)
Family/friend provided interpretation	26 (65%)	0 (0%)
Interpretation services used	3 (8%)	0 (0%)
No interpreter used	11 (27%)	3 (100%)

Four qualitative themes were identified among those with non-English preference as summarized in **Table 4**. These included the use of workarounds to access interpretation using family or other just-in-time solutions, frustration about language barriers, and calling for the need for improved access to healthcare services in their language.

**Table 4:** Emergent qualitative themes among interviews of non-English preferred patients

Theme	Description	Participant quotes
Family member interpretation support	Family members and friends provided significant support assisting non-English speaking patients with understanding and adhering to instructions	“If (the patient) didn’t have someone to help him, he would not be able to understand the instructions as his comprehension in English is not good enough to understand medical terms”. “If patients don’t have family around, understanding and following instructions would be challenging for them”.
Just-in-time solutions	Translation was often accessed via non-hospital provided services (e.g., Google translate, a healthcare worker who speaks the patient’s language, etc.)	“Some nurses were using Google translate with me, which was helpful”.
Patient frustration with language barrier	Patients expressed communication challenges with their care teams despite availability and use of existing hospital-provided translation services	“If I was able to speak English, my experience would have been better”. “I was very upset because I couldn’t communicate how I was feeling until the nurse who spoke my language came to help”.
Patient and family ideas for improvement	Suggested services and options to enhance the experience of patients who prefer non-English languages	“I would like to see written documentation given to [non-English speaking] patients in their primary languages, as is done in the local school system for parents”. “If family members are not here, it would be helpful for patients to know what options exist for translation and how to access those services”.

## Discussion

In this Canadian study examining the effect of language preference on understanding and adhering to hospital infection prevention protocols, we identified no significant difference among non-English preferred patients; however, addressing this gap relied on family members or other informal solutions. This finding is supported by both the quantitative and qualitative data, suggesting that existing hospital-based interpretation services were infrequently used in these situations.

Disparities in access to language concordant care exist across Canada and have been shown to influence patient outcomes, including repeat hospital visits, readmissions and in-hospital death ((7,8)). In a retrospective cohort study of homecare recipients admitted to hospital in Ontario, Canada, those who received over 50% of their care from physicians who spoke the patients' primary language had better in-hospital outcomes ((8)).

The effect of language concordant care on development of healthcare-associated infection in Canadian hospitals is far less clear. One important barrier is that data related to patients’ preferred language and data related to health equity are not routinely recorded on admission to hospital or linked to hospital infection surveillance, as practiced in other countries ((3)). Self-care of vascular catheters and preoperative bathing before cardiac surgery are both essential practices that have been shown to reduce the risk of healthcare-associated infection ((10,11)). These practices were therefore well suited to systematically assess the impact of language preference on implementing these critical infection prevention instructions in Canadian hospitals.

While an overall difference was not identified in comprehension or adherence, language concordant delivery of infection prevention protocols frequently relied on workarounds in our hospitals. Despite the small number (n=3), 100% of those who did not comprehend instructions had no interpretation provided, which provides a glimpse into the inequity that exists if family or other informal means of interpretation is not present to fill this gap. In addition, relying on family or other informal solutions for interpretation of critical information for patients is associated with potential risks of misinterpretation due to the lack of formal training ((4)). The qualitative data in our study supports the idea that patients and family members expressed frustration with having to fulfill this role and wished for improved hospital-based interpretation services. In response to these and other patient experience data, the two organizations capture language preference on admission and have increased utilization of hospital-based interpretation via a mobile system.

The study has several important strengths. It was conducted across two acute care hospitals which improves the external validity of these findings. A validation of the questionnaire was performed to ensure that it was reliable between interviewers and assessed for confounders including use of informal interpretation solutions. Both closed and open-ended questions were included to fully explore patient and family views. Finally, patients were surveyed in two different patient populations receiving different types of infection prevention protocols.

### Limitations

Several important limitations should be noted. First, the study relied on self-reported adherence to infection prevention protocols, which may overestimate actual adherence due to recall bias. Second, a small number of interviews could not be completed due to unavailability of specific language interpreters, which could have differentially excluded non-English preference participants. Third, both participating hospitals are located in a predominantly English speaking region, whereas results may differ in other parts of Canada where French or Indigenous languages are more prevalent. In addition, the large urban setting has a large variety of non-English languages that has likely prompted increased focus on language concordant care compared to other Canadian hospitals in smaller or non-urban settings.

## Conclusion

Although the study did not detect a significant difference in understanding and reported adherence based on language preference, it highlighted the risks and inequity of having to rely on family members or other informal solutions. Hospitals should focus on encouraging the use of formal interpretation services when delivering critical infection prevention instruction.
